# Advantages of an Electrochemical Method Compared to the Spectrophotometric Kinetic Study of Peroxidase Inhibition by Boroxine Derivative

**DOI:** 10.3390/molecules22071120

**Published:** 2017-07-05

**Authors:** Jelena Ostojić, Safija Herenda, Zerina Bešić, Mladen Miloš, Borivoj Galić

**Affiliations:** 1 Department of Chemistry, Faculty of Science, University of Sarajevo, Zmaja od Bosne 33-35, 71 000 Sarajevo, Bosnia and Herzegovina; islamovic.safija@gmail.com (S.H.); zerinaa93@hotmail.com (Z.B.); borivoj.galic@gmail.com (B.G.); 2 Faculty of Chemistry and Technology, University of Split, Teslina 10, 21 000 Split, Croatia; milos@ktf-split.hr

**Keywords:** boroxine derivative, peroxidase, enzyme kinetics, cyclic voltammetry

## Abstract

In this study, boroxine derivative (K_2_[B_3_O_3_F_4_OH]) was tested as an inhibitor of horseradish peroxidase (HRP) by spectrophotometric and electrochemical methods. The activity of horseradish peroxidase was first studied under steady-state kinetic conditions by a spectrophotometric method which required the use of guaiacol as a second substrate to measure guaiacol peroxidation. The results of this method have shown that, by changing the concentration of guaiacol as the literature suggests, a different type of inhibition is observed than when changing the concentration of hydrogen peroxide as the substrate. This suggests that guaiacol interferes with the reaction in some way. The electrochemical method involves direct electron transfer of HRP immobilized in Nafion nanocomposite films on a glassy carbon (GC) electrode, creating a sensor with an electro-catalytic response to the reduction of hydrogen peroxide. The electrochemical method simplifies kinetic assays by removing the requirement of reducing substrates.

## 1. Introduction

The boroxine derivative, dipotassium-trioxohydroxytetrafluorotriborate K_2_[B_3_O_3_F_4_OH], has lately been intensively studied as a potential enzyme inhibitor. K_2_[B_3_O_3_F_4_OH] has been shown to be an effective inhibitor of catalase and some human carbonic anhydrases [1,2]. Most recent research has investigated K_2_[B_3_O_3_F_4_OH] as an inhibitor of horseradish peroxidase (HRP) and has found that K_2_[B_3_O_3_F_4_OH] binds to the native HRP enzyme, showing an example of competitive inhibition [3].

Horseradish peroxidase is an important heme-containing enzyme and is widely used for analytical purposes, biosensors and enzymatic bioreactors [4,5,6].

The detection of HRP activity is widely used in labelling systems and a large number of procedures for monitoring its reaction have been developed. To monitor HRP activity, substances often referred to as peroxidase substrates need to be added to the reaction mixture. These substrates are electron donors and, when oxidized by HRP with hydrogen peroxide present as the oxidizing agent, a characteristic change that is detectable by the spectrophotometric method takes place [7,8]. The most common substrate used as an electron donor is guaiacol; it was therefore also used in this research. A spectrophotometric assay of peroxidase activity using guaiacol is a common analytical method for quantifying enzymatic activity based on the change in absorbance at 470 nm. The formed product, tetraguaiacol, is amber-colored and is detectable by a UV/VIS spectrophotometer [9,10,11,12,13].

Nowadays, there is also an increasing trend in making biosensors which include immobilized HRP for the removal of hydrogen peroxide. The activity of HRP when immobilized can be monitored using chronoamperometry and cyclic voltammetry can be used for the characterization of the immobilized enzyme. Electrochemical techniques have their advantages because of their simplicity, low cost and speed. The only condition for this method of monitoring enzyme kinetics is that direct electron transfer must be possible or able to be achieved by mediators [14,15,16,17,18]. Efficient electron transfer is difficult to achieve in many cases because the active site of an enzyme can be trapped deep inside the enzyme structure, as well as because denaturation of the enzyme can easily occur during the process of immobilization. Direct electron transfer of HRP has been reported in many papers using many carriers: for example, sol-gel matrices, biopolymers, and nanomaterials [19,20,21,22,23]. Many enzymes including HRP have been immobilized using a Nafion matrix because it does not show any denaturalization of enzymes or their enzymatic activity.

Direct electron transfer of immobilized HRP is based on the Fe(III)/Fe(II) reduction in the active heme center of the enzyme.

When H_2_O_2_ is involved, the reaction mechanism is a little more complex. HRP reacts with H_2_O_2_ and forms an oxidized form of the enzyme called Compound I (HRP (Fe^+4^=O)^+^), which is actually an intermediate. Compound I contains oxyferryl heme (Fe^+4^=O) and a porphyrin π cation radical. Compound I is also catalytically active and, when receiving one electron from the substrate, converts to Compound II (HRP (Fe^+4^=O)). Compound II also contains an Fe(IV) oxyferryl species that is one oxidizing equivalent above the resting state. The catalytic mechanism is shown below in steps:

HRP (Fe-III) + H_2_O_2_ → HRP (Fe^+4^=O)^+^ + H_2_O (k_1_) (1)

HRP (Fe^+4^=O)^+^ + AH_2_ → HRP (Fe^+4^=O) + AH**∙** (k_2_) (2)

HRP (Fe^+4^=O) + AH_2_ → HRP (Fe-III) + AH + H_2_O (k_3_) (3)

AH**∙** + e^−^ + H^+^ → AH_2_ + H_2_O (k_4_) (4)

where HRP (Fe-III) is the native enzyme, sometimes referred as the resting enzyme, HRP (Fe^+4^=O)^+^ and HRP (Fe^+4^=O) are the oxidized forms of the enzyme and AH_2_ and AH**∙** are the electron donor substrate and the radical product of its one-electron oxidation [24].

The enzyme immobilized on an electrode when hydrogen peroxide is present is being oxidized and converted to Compound I, as shown in reaction (1). Compound I is further reduced by electrons provided from an electrode, reaction (5):

HRP (Fe^+4^=O)^+^ + 2e^−^ + 2H^+^ → HRP (Fe-III) + H_2_O (k_s_) (5)

Electron donors (AH_2_ from reaction 2 and 3) are unnecessary, because the electrode provides both electrons and the enzyme is converted in its resting state.

An electron donor can also be present in a peroxidase–electrode system; both processes can then occur at the same time and the oxidized donor AH**∙** is then electrochemically reduced by an electrode, as shown in reaction (4). The other case is known as a mediated electron transfer, which is sometimes more efficient than direct electron transfer.

The purpose of this paper is to show that the electrochemical method has advantages over the spectrophotometric method; mostly because of the ease of conducting experimental work. The other reason is that most literature recommends the use of guaiacol for spectrophotometric measurements; not only to be present in the reaction system, but also to use it when changing the concentration of the substrate, because it avoids increasing H_2_O_2_ concentration to prevent the inactivation of the enzyme. For that reason, we conducted the same experiments and untypical behavior of our inhibitor was shown. Furthermore, the goal of our research was to compare two methods and to show that, when using the spectrophotometric method, electron donors such as guaiacol could interfere with the inhibitor K_2_[B_3_O_3_F_4_OH] and cause problems when determining the type of inhibition.

## 2. Results

### 2.1. Determination of K_m_ and V_max_—Spectrophotometric Method

For kinetic measurements using the spectrophotometric method with no inhibitor present, the kinetic constants calculated were: K_m_ = 0.47 mM and V_max_ = 0.34 mM min^−1^ when guaiacol was only present in a fixed concentration (1.33 mM) and where plots were gained as a function of concentration of H_2_O_2_.

**Figure 1:**
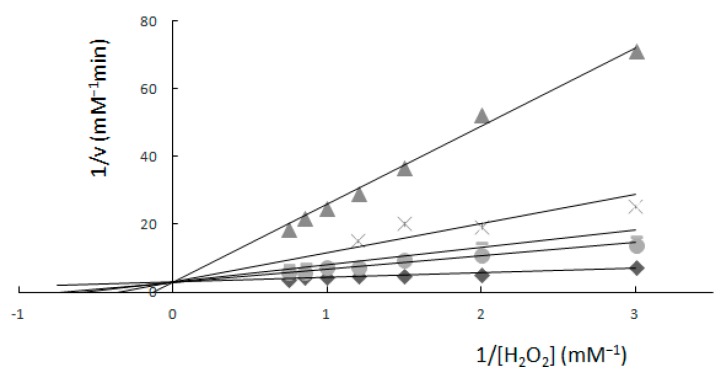
The graphical determination of kinetic constants from Lineweaver–Burk plots with a fixed concentration of guaiacol (1.33 mM). Fixed concentrations of K_2_[B_3_O_3_F_4_OH] were: (◊) 0.0 mM, (○) 4 mM, (-) 6 mM, (×) 10 mM, (Δ) 39.6 mM. Results gained by the spectrophotometric method. Reprinted from reference [3].

When an inhibitor was present, the same kinetic measurements were performed and, using a double reciprocal graph, the calculated value of V_max_ was 0.34 mM min^−1^, but K_m_ values were different for each concentration of K_2_[B_3_O_3_F_4_OH]. Km values were 1.35, 1.76, 2.92, 6.48 and 7.85 mM with respect to the concentration of K_2_[B_3_O_3_F_4_OH] (Figure 1).

For kinetic measurements when H_2_O_2_ concentration was kept fixed (0.32 mM), Lineweaver–Burk plots were gained as a function of guaiacol concentration; the calculated kinetic constants were: K_m_ = 3.87 mM, V_max_ = 0.57 mM min^−1^ with no inhibitor present.

**Figure 2:**
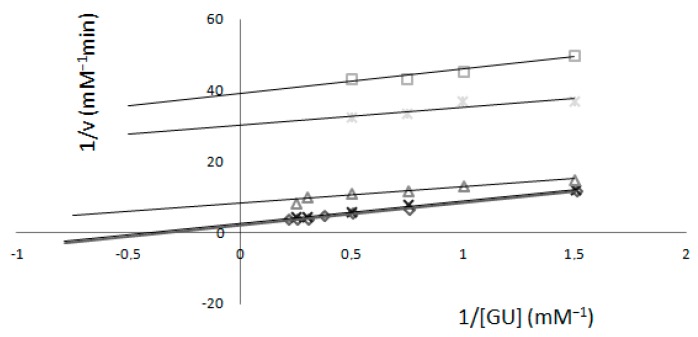
The graphical determination of kinetic constants from Lineweaver–Burk plots with a fixed concentration of H_2_O_2_ (0.32 mM). Fixed concentrations of K_2_[B_3_O_3_F_4_OH] were: (◊) 0.0 mM, (×) 0.3 mM, (Δ) 2.0 mM, (Ж) 4.00 mM, (□) 6.0 mM. Results gained by the spectrophotometric method.

When an inhibitor was present, values of K_m_ and V_max_ were different relative to the concentration of K_2_[B_3_O_3_F_4_OH]. Calculated K_m_ values were 3.05, 2.41, 0.41, 0.17 and 0.17 mM, and from the intersection of the y-axis, the V_max_ values were also variable: 0.49, 0.38, 0.10, 0.03 and 0.02 mM min^−1^ relative to the concentration of K_2_[B_3_O_3_F_4_OH] (Figure 2).

### 2.2. Determination of K_m_ and I_max_—Electrochemical Method

**Figure 3:**
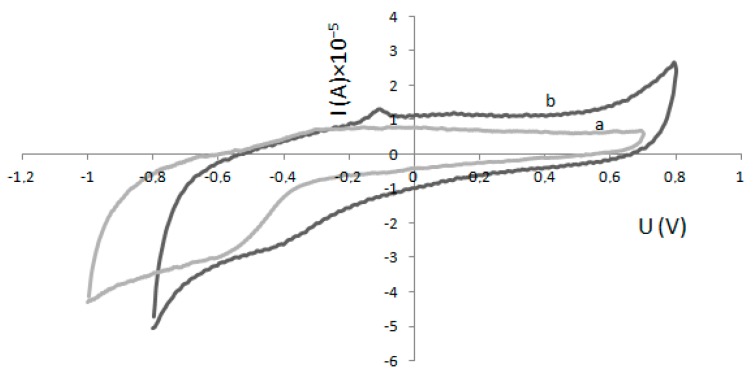
Cyclic voltammograms of a glassy carbon (GC) electrode modified with Nafion (curve b) and a GC electrode modified with horseradish peroxidase (HRP) and Nafion (curve a) in 100 mM phosphate buffer solution (PBS pH 6.0) at a scan rate of 50 mV s^−1^.

As mentioned before, electrochemical measurements need no involvement from the electron donor substrate, yet it can be used in the reaction system, which then becomes a mediated electron transfer. Curve b (Figure 3) shows the cyclic voltammogram of a GC electrode modified only with Nafion, and curve a (Figure 3) shows the cyclic voltammogram of a GC electrode modified with Nafion and enzyme (HRP) film. Cyclic voltammetry measurements were performed in 100 mM phosphate buffer solution (PBS pH 6.0) at a scan rate of 50 mV s^−1^. Pairs of redox peaks were established at the HRP/Nafion composite film (curve a) from the electrochemical reaction between HRP-Fe(III) and HRP-Fe(II). Redox peaks were not established on the cyclic voltammogram of a GC electrode modified only with Nafion. The anodic peak potential (E_pa_) was observed at 0.31 V and the cathodic peak potential (E_pc_) was at 0.41 V. This confirms that HRP film with Nafion formed on a GC electrode can perform direct electron transfer of HRP. When H_2_O_2_ is added, it should react with HRP-Fe(III), forming O_2_ and causing an increase in the current of these two reduction peaks.

**Figure 4:**
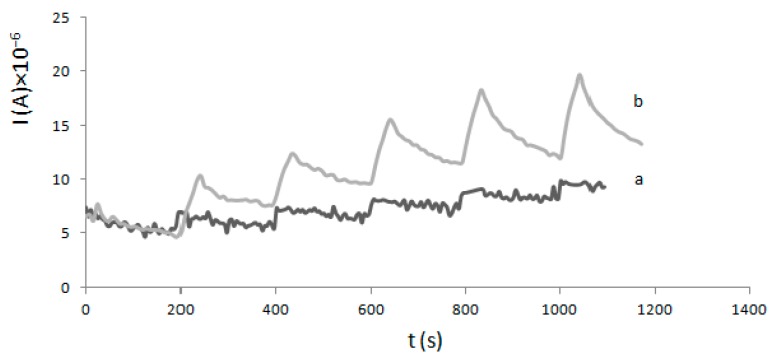
Amperometric responses of a GC electrode modified with HRP and Nafion (b) and a GC electrode modified only with Nafion (a) upon successive additions of H_2_O_2_ in 100 mM phosphate buffer solution (PBS pH 6.0).

Figure 4 shows the amperometric responses of both previously described electrodes upon successive additions of H_2_O_2_ in 100 mM phosphate buffer (pH 6.0) at an applied potential of +0.9 V. Amperometric curves showed that the current increases after the addition of H_2_O_2_ at a GC electrode with enzyme/Nafion but has almost no response at electrodes modified only with Nafion.

As is already known, the apparent Michaelis–Menten constant (K_m_) can be calculated from the Lineweaver–Burk equation [25]:

1/I_ss_ = 1/I_max_ + K_m_/I_max_ 1/C;

I_ss_ is the steady-state current after the addition of substrate, C is the bulk concentration of substrate and I_max_ is the maximum current measured under saturated substrate solution.

The Michaelis–Menten constant (K_m_) is calculated from the slope and interception of the reciprocal plot of the steady-state current vs. H_2_O_2_ concentration.

The Michaelis–Menten constant (K_m_) calculated in this work in the absence of an inhibitor was 33.1 mM, suggesting that the HRP and Nafion modified glassy carbon electrode shows a lower affinity for H_2_O_2_ than usually reported in similar papers and also a lower affinity than for the native enzyme. The kinetic constants (e.g., K_m_, V_max_) of immobilized enzymes are usually different from the kinetic constants of free enzymes, which is most often explained by internal structural changes and confined access to the enzyme active site. This may be due to changes in the properties of the solution in the region near the immobilized enzyme or the effects of molecular diffusion within the local environment.

**Figure 5:**
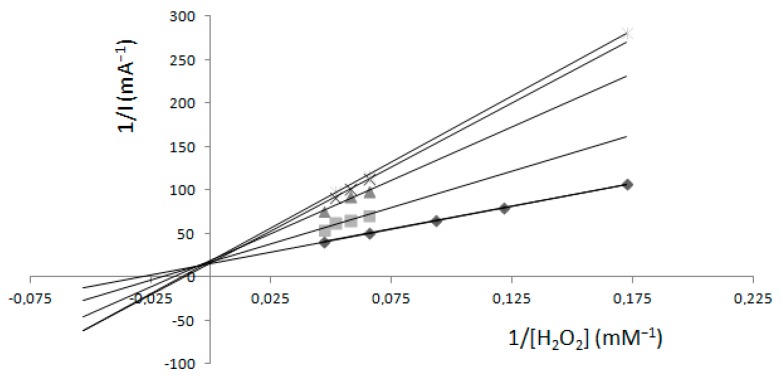
The graphical determination of kinetic constants from Lineweaver–Burk plots without guaiacol present. Fixed concentrations of K_2_[B_3_O_3_F_4_OH] were: (◊) 0 mM, (□) 2 mM, (Δ) 4 mM, (×) 6 mM, (Ж) 10 mM; Results gained by the electrochemical method.

To determine the inhibition effect of K_2_[B_3_O_3_F_4_OH], electrochemical measurements (Figure 5) were performed at fixed concentrations of K_2_[B_3_O_3_F_4_OH] as a function of H_2_O_2_ concentration. From a Lineweaver–Burk plot, the calculated I_max_ was 0.06 mA. From the same plot, the calculated K_m_ values were 33.1, 48.2, 63.4, 89.1 and 79.7 mM, relative to concentration of K_2_[B_3_O_3_F_4_OH].

**Figure 6:**
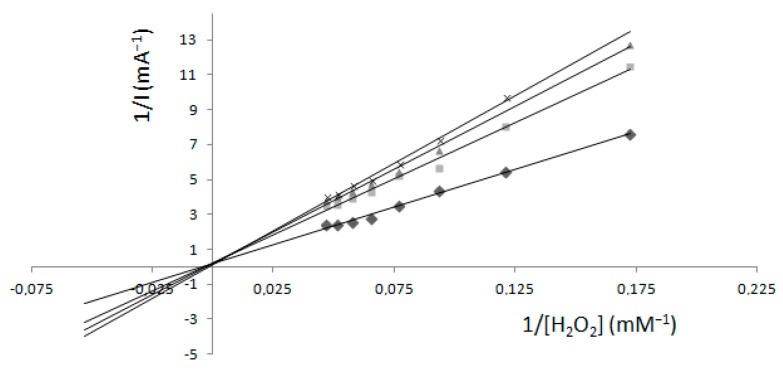
The graphical determination of kinetic constants from Lineweaver–Burk plots with guaiacol present in a fixed concentration of 1.33mM. Fixed concentrations of K_2_[B_3_O_3_F_4_OH] were: (◊) 0 mM, (□) 4 mM, (Δ) 8 mM, (×) 10 mM; Results gained by the electrochemical method.

Electrochemical measurements (Figure 6) when guaiacol was present in an electrochemical cell in a concentration of 1.33 mM were also performed at a fixed concentration of K_2_[B_3_O_3_F_4_OH] as a function of H_2_O_2_ concentration. From a Lineweaver–Burk plot, a unique I_max_ = 5.4 mA was calculated and K_m_ values were variable at 214.6, 294.3, 380.3 and 526 mM, relative to concentration of K_2_[B_3_O_3_F_4_OH].

## 3. Discussion

### Comparison of Two Methods

K_2_[B_3_O_3_F_4_OH] was tested as an inhibitor of HRP activity under steady-state kinetic conditions after the preincubation of the enzyme with different concentrations of K_2_[B_3_O_3_F_4_OH]. Guaiacol oxidation by HRP was used to evaluate the enzymatic activity and to test the influence of the inhibitor.

The assays for guaiacol oxidation were carried out with measurements as a function of H_2_O_2_ concentrations and as a function of guaiacol concentrations, showing different results.

The measurements in relation to hydrogen peroxide (Figure 1) showed that values of V_max_ stayed unchanged at each K_2_[B_3_O_3_F_4_OH] concentration. The effect of K_2_[B_3_O_3_F_4_OH] on H_2_O_2_ oxidation of guaiacol as an electron donor showed that K_2_[B_3_O_3_F_4_OH] acts as a competitive inhibitor for the enzyme. As the concentration of K_2_[B_3_O_3_F_4_OH] was increased, the apparent Km value also increased; the data are consistent with competitive inhibition.

The measurements in relation to guaiacol (Figure 2) showed changes in both values of V_max_ and K_m_; the data are consistent with uncompetitive inhibition suggesting that K_2_[B_3_O_3_F_4_OH] induced conformational changes and affects both peroxide sites and substrate sites.

Since the results with the spectrophotometric method were different for two forms of measurements, we performed the electrochemical method to compare the results. Electrochemical measurements were also carried out in two ways. The first form used only H_2_O_2_ with an immobilized electrode and, for the second, guaiacol was present in the electrochemical cell as a mediator. The electrochemical method (Figure 5 and Figure 6) in both forms showed that values of I_max_ remained unchanged at each K_2_[B_3_O_3_F_4_OH] concentration, pointing to competitive inhibition. These results agreed with spectrophotometric results in relation to H_2_O_2_ where the concentration of guaiacol was kept fixed.

The lower signal (mA) in the absence of guaiacol (Figure 5) and the lower K_m_ value in comparison with measurements in the presence of 1.33 mM guaiacol (Figure 6) is probably due to the slow electron transfer, which can be improved by adding a mediator to transfer electrons between the electrode surface and the hydrogen peroxide [26,27]. Slow electron transfer is also affected by operational parameters, such as the operating potential, mediator concentration and pH, and thermal stability. These parameters are very important when searching for the best analytical performance when using an electrode as a sensor.

The electrochemical method proved here to be very useful to test the effect of new inhibitors. The most important aspect is that it is in correlation with the more commonly-used spectrophotometric method, yet is much simpler and requires less time to follow the enzymatic activity of horseradish peroxidase under the influence of possible inhibitors.

This study also showed that an electrochemical biosensor could be prepared without a mediator for the purpose of following enzymatic activity, regardless of the lower Km value gained. This is very important because direct electrochemical behavior of the enzyme or protein at the electrode simplifies the preparation processes of biosensors.

## 4. Materials and Methods

### 4.1. Chemicals

K_2_[B_3_O_3_F_4_OH] is a boron inorganic derivative synthesized by reacting potassium hydrofluoride (KHF_2_) with boric acid, working in molar ratios of 2:3 at room temperature as reported in the literature [28]. All other compounds used were obtained as highest-purity reagents from Sigma Aldrich (Saint Louis, MO, USA) and Fisher Chemical (Waltham, MA, USA).

### 4.2. Spectrophotometric Assay of HRP Activity

Guaiacol peroxidation was monitored spectrophotometrically by following the increase of the absorbance at 470 nm of the reaction mixtures within the first few minutes. The method was described by Chance and Maehly [29]. The instrument used was a MultiskanGOMicroplate spectrophotometer, Thermo Fisher Scientific, Waltham, MA, USA.

For hydrogen peroxide kinetic measurements, guaiacol concentration (1.33 mM) was kept fixed and H_2_O_2_ concentration varied between 0.33 and 1.33 mM. Assays were carried out at a temperature of 25 °C. The total volume of reaction mixture was 300 µL, consisting of phosphate buffer at pH 6.0 (100 mM), 25 μL of enzyme solution (approximately 0.45 nM, assuming the molecular weight of HRP as 44 kDa) and 25 µL of guaiacol. Volume of H_2_O_2_ was varied to make different concentrations.

When testing inhibition, the inhibitor was also present in the reaction mixture and in every experiment inhibitor was preincubated for 5 minutes with the enzyme before each measurement. The reaction was started after the addition of H_2_O_2_.

For guaiacol kinetic measurements, H_2_O_2_ concentration (0.42 mM) was kept fixed and guaiacol concentration varied between 0.6 mM to 4.6 mM. As in the case of kinetic measurements for hydrogen peroxide, measurements were also conducted with different concentrations of inhibitor under the same conditions. The volume of guaiacol was varied in this case to make the above-mentioned concentrations.

### 4.3. Electrode Modification and Electrochemical Experiments

Cyclic voltammetry and amperometric experiments were carried out on PAR 263A potentiostat/galvanostat and the usual three-electrode system. A saturated Ag/AgCl electrode was employed as a reference electrode, a Pt electrode as a counter electrode and a modified glassy carbon (GC) electrode as the working electrode.

All measurements were carried out in 100 mM pH 6.0 phosphate buffer solution (PBS) and conducted at room temperature. A magnetic stirrer (approximately 400 rpm) was employed during the amperometric measurements.

The electrochemical characterization of the immobilized enzyme was done by cyclic voltammetry.

Before the immobilization, the GC electrode surface was polished with 0.05 mm Al_2_O_3_ and ultrasonically cleaned with acetone—NaOH (1:1), HNO_3_ (1:1)—and rinsed with double distilled water. Then the modified electrode was left to dry at room temperature.

After cleaning the GC electrode, a volume of 10 µL of a mixed solution of 0.05 ng/mL HRP and 5% (*w*/*w*) Nafion was dripped on the surface of the electrode. The HRP/Nafion electrode was left to dry at room temperature for at least 90 minutes.

Amperometric measurements were carried out in the electrochemical cell containing 25 mL of buffer solution. The modified electrode, together with the reference and the counter electrode, was immersed into a phosphate buffer solution and the change of current was observed when 0.1 mM H_2_O_2_ was added in the mixture. A constant potential of 0.9 V was applied on the working electrode. The reaction was monitored in the absence and in the presence of different concentrations of K_2_[B_3_O_3_F_4_OH].

## 5. Conclusions

This study showed that the enzyme horseradish peroxidase follows the Michaelis–Menten kinetic model, in the absence and in the presence of the inhibitor K_2_[B_3_O_3_F_4_OH]. The presence of the inhibitor K_2_[B_3_O_3_F_4_OH] increases the Michaelis constant K_m_ without an impact on the maximum rate V_max_ (I_max_) of enzyme reaction. Results suggested that K_2_[B_3_O_3_F_4_OH] is a classical competitive inhibitor. Results from the spectrophotometric and electrochemical methods without guaiacol as a substrate were in agreement, both indicating competitive inhibition. The spectrophotometric method in the presence of varied concentrations of guaiacol showed uncompetitive inhibition which can lead to the conclusion that guaiacol in higher concentrations interferes with the reaction in some way. Therefore, the electrochemical method is more suitable for the measurement of kinetic parameters, since there is no need for the electron donor guaiacol to be present.
